# Effectiveness of eHealth Interventions Targeting Employee Health Behaviors: Systematic Review

**DOI:** 10.2196/38307

**Published:** 2023-04-20

**Authors:** Aleksandra Sevic, Neda S Hashemi, Mikkel Magnus Thørrisen, Kine Strømstad, Lisebet Skeie Skarpaas, Marianne Storm, Kolbjørn Kallesten Brønnick

**Affiliations:** 1 Department of Public Health Faculty of Health Sciences University of Stavanger Stavanger Norway; 2 Department of Rehabilitation Science and Health Technology Faculty of Health Sciences OsloMet – Oslo Metropolitan University Oslo Norway; 3 Faculty of Health Sciences and Social Care Molde University College Molde Norway; 4 Department of Quality and Health Technology Faculty of Health Sciences University of Stavanger Stavanger Norway

**Keywords:** eHealth, employees, workplace, health behaviors, sickness absence, behavior change techniques, systematic review, mobile phone

## Abstract

**Background:**

The number of people with noncommunicable diseases is increasing. Noncommunicable diseases are the major cause of disability and premature mortality worldwide, associated with negative workplace outcomes such as sickness absence and reduced work productivity. There is a need to identify scalable interventions and their active components to relieve disease and treatment burden and facilitate work participation. eHealth interventions have shown potential in clinical and general populations to increase well-being and physical activity and could be well suited for workplace settings.

**Objective:**

We aimed to provide an overview of the effectiveness of eHealth interventions at the workplace targeting employee health behaviors and map behavior change techniques (BCTs) used in these interventions.

**Methods:**

A systematic literature search was performed in PubMed, Embase, PsycINFO, Cochrane CENTRAL, and CINAHL in September 2020 and updated in September 2021. Extracted data included participant characteristics, setting, eHealth intervention type, mode of delivery, reported outcomes, effect sizes, and attrition rates. Quality and risk of bias of the included studies were assessed using the Cochrane Collaboration risk-of-bias 2 tool. BCTs were mapped in accordance with the BCT Taxonomy v1. The review was reported according to the PRISMA (Preferred Reporting Items for Systematic Reviews and Meta-Analyses) checklist.

**Results:**

In total, 17 randomized controlled trials met the inclusion criteria. The measured outcomes, treatment and follow-up periods, content of eHealth interventions, and workplace contexts had high heterogeneity. Of the 17 studies, 4 (24%) reported unequivocally significant findings for all primary outcomes, with effect sizes ranging from small to large. Furthermore, 53% (9/17) of the studies reported mixed results, and 24% (4/17) reported nonsignificant results. The most frequently targeted behavior was physical activity (15/17, 88% of the studies); the least frequently targeted behavior was smoking (2/17, 12% of the studies). Attrition varied greatly across the studies (0%-37%). Risk of bias was high in 65% (11/17) of the studies, with some concerns in the remaining 35% (6/17). Interventions used various BCTs, and the most frequently used were *feedback and monitoring* (14/17, 82%), *goals and planning* (10/17, 59%), *antecedents* (10/17, 59%), and *social support* (7/17, 41%).

**Conclusions:**

This review suggests that, although eHealth interventions may have potential, there are still unanswered questions regarding their effectiveness and what drives the mechanism behind these effects. Low methodological quality, high heterogeneity and complexity, the characteristics of the included samples, and often high attrition rates challenge the investigation of the effectiveness and the making of sound inferences about the effect sizes and significance of the results. To address this, new studies and methods are needed. A megastudy design in which different interventions are evaluated in the same population over the same period on the same outcomes may solve some of the challenges.

**Trial Registration:**

PROSPERO CRD42020202777; https://www-crd-york-ac-uk/prospero/display_record.php?RecordID=202777

## Introduction

### Background

Noncommunicable diseases (NCDs) are the major cause of avoidable disability and premature mortality worldwide and are considered a global health priority by the World Health Organization (WHO) [1]. The WHO estimates that over a billion people worldwide live with disability and that NCDs are the cause behind two-thirds of all years lived with disability [1]. Negative workplace outcomes such as sickness absence, reduced work performance, and work cessation are often associated with health issues caused by NCDs [2-5].

Health behaviors such as smoking, alcohol use, poor diet, and physical inactivity may considerably increase one’s risk of NCDs [6-10]. Up to an estimated 80% of cases of heart disease, stroke, and type 2 diabetes and >40% of cancers worldwide may be prevented through behavior change [11]. In addition to being linked to poor health outcomes, these health behaviors have also been linked to poor work participation outcomes such as sickness absence [12-16], decreased work productivity [17,18], and increased financial costs for employers [19-21]. Health behavior change may considerably reduce some of the burden of NCDs and NCD-caused disability and subsequently increase work ability and work performance and reduce sickness absence [5]. Therefore, efforts should be focused on disease prevention and health behavior promotion through behavior change, such as increasing physical activity (PA), reducing alcohol consumption, quitting smoking, and eating a healthy diet, as well as weight management [22].

The workplace is a major arena for health interventions and workplace health promotion programs (WHPP) aiming at preventing NCDs and promoting positive health-related behaviors [5,23]. Workers spend a substantial proportion of their waking hours at the workplace, making it both viable and practical to implement health behavior interventions there [5,24,25]. Ample research in this field has reported positive evidence of the effectiveness of WHPPs [25-27]. However, systematic reviews have identified some of the challenges of WHPPs, such as small effects [5] and considerable heterogeneity, which makes drawing clear conclusions and generalizations of findings about the effects of these programs challenging [28]. Rongen et al [5] stated that the effectiveness of WHPPs hinges on the robustness of the study design and methods, characteristics of the population, and intervention content. Thus, although there are many potential benefits in implementing WHPPs and interventions, unraveling the complexity of these interventions and the consequences it might have on their effectiveness is important to facilitate implementation, generalizability of the findings, and systematic evaluation.

Since the early 90s, there has been a tremendous increase in the delivery of eHealth interventions aimed at behavior change, health promotion, disease and disability prevention, and returning to work [29-31]. eHealth combines the use of technologies such as the internet, smartphones, and activity trackers to aid behavior change, promote health, and reduce sickness absence [32]. The benefits attributed to eHealth are many, such as extending the scope, availability, and reach of health care; empowering end users; improving self-management; personalizing health services; providing anonymity in some cases; and facilitating communication [33]. Extensive research has been conducted on the topic of the effectiveness of eHealth interventions targeting health behaviors in the general population [34], and there is some evidence of the short-term effectiveness of eHealth interventions [32] and recently on the long-term effectiveness of exclusively internet-delivered interventions [35]. However, there is insufficient evidence regarding the long-term effectiveness of eHealth interventions in general and a need for more research exploring the reach, use, and engagement; effectiveness in diverse groups; and the active components used in these interventions [32,34,35].

Regarding eHealth interventions targeting employees, Howarth et al [36] published a review of 22 randomized controlled trials (RCTs) focusing on the impact of purely digital interventions on health-related outcomes in the workplace. The authors included studies that targeted physical, psychological, biological, behavioral, and work measures. They found limited evidence that digital-only interventions have a positive influence on health-related outcomes such as sleep, mental health, and PA levels. The authors [36] suggested that digital interventions are a promising method for improving employee health. However, further research is needed to distinguish which interventions work best for different health outcomes and which active components or behavior change techniques (BCTs) are used in these interventions.

Although expectations regarding the use of eHealth may be great, unknowns are perhaps even greater, and the effects are often ambiguous because of the complexity of eHealth interventions and the heterogeneity surrounding the implementation contexts, study populations, and outcomes.

The previous paragraphs point toward the lack of evidence regarding some aspects of eHealth interventions as well as the importance of complexity in eHealth interventions and WHPPs. The Cochrane Handbook for Systematic Reviews of Interventions [37] defines 3 ways in which interventions may be seen as complex. They are as follows: (1) the intervention itself may be complex and may have many components, (2) the intervention may lead to complex interactions, and (3) the context or systems in which the intervention is being implemented may be complex. In this review, we primarily focused on the complexity of the intervention itself. To achieve this and understand the mechanism behind the effect of eHealth interventions, we mapped the active components of the interventions. The BCT Taxonomy v1 (BCTTv1) developed by Michie et al [38] is a method for mapping BCTs—the smallest active components of health interventions designed to instigate or alter individuals’ behavior. The taxonomy has previously been used to map BCTs in apps [39,40]. As Michie et al [38] point out, health interventions that aim for behavior change are often complex, which in turn “makes them challenging to replicate in research, to implement in practical applications, and to synthesize in systematic literature reviews” [38]. Therefore, mapping the smallest active components of these interventions would potentially aid in understanding what drives the effects of these interventions and the mechanisms behind them.

### Objectives

Thus, NCDs may be considerably reduced by improving health behaviors [8,11]. Targeting employees may reduce sickness absence and presenteeism, increase work productivity, and improve the long-term health of employees [12,41,42]. However, presently, there is a lack of systematic reviews investigating the effectiveness of eHealth interventions targeting employee health behaviors. It is still unclear whether eHealth interventions are effective for different health behaviors in this population. Furthermore, a thorough overview of the BCTs used in such interventions in the employee population is currently lacking. Hence, the aim of this review was to provide an overview of the effectiveness of eHealth interventions on the health behaviors of employees and map the BCTs used in these interventions, which may conceivably lead to a greater understanding of what constitutes an effective eHealth intervention for this population.

## Methods

### Protocol and Registration

The review was designed and conducted in accordance with the Cochrane recommendations [37] and followed the PRISMA (Preferred Reporting Items for Systematic Reviews and Meta-Analyses) guidelines [43]. The review was registered in the PROSPERO database (CRD42020202777).

### Eligibility Criteria

Studies were included if the criteria outlined in Textbox 1 were fulfilled.

**Inclusion criteria.**

*Type of participants*: employees aged ≥18 years (fully, partially, or self-employed)

*Type of intervention*: eHealth was a major component of the intervention (delivered via a smartphone, computer, tablet, or wearable activity monitor or tracker either via email, app, website, or a software)

*Type of control group*: intervention included a nondigital control group (waitlist, care as usual, or active control)

*Type of outcome measures*: those that measured one or more health behaviors as primary outcomes, such as the following:

- Smoking (reduction in or abstinence from smoking)
- Alcohol consumption (Timeline Followback [44], Alcohol Use Disorders Identification Test [45], Fast Alcohol Screening Test [46], or any other relevant measure)
- Diet (reduction in food intake or adherence to a healthier diet or any other relevant measure reported)
- Physical activity (measures of physical activity using portable devices, such as steps per day or time spent in moderate to vigorous physical activity per day or week and other relevant measures reported), including sedentary behaviors

*Study design*: randomized controlled trials (RCTs; excluding cluster RCTs and including pilots)

*Type of publication*: full-text research article published in a scientific peer-reviewed journal

*Language*: studies published in English or a Scandinavian language (Norwegian, Swedish, or Danish)

*Time*: published between 1990 and 2021.

To lower the heterogeneity and possible biases from randomization at a different unit level, we excluded cluster RCTs and included only those studies that used individuals as units of randomization [47,48].

### Search Strategy and Data Screening

The search terms were developed in consultation with an experienced librarian. Searches were conducted in the following 5 databases: PubMed, Embase, PsycINFO, Cochrane CENTRAL, and CINAHL. The initial search was performed in September 2020 for the period from 1990 to the present time. The year 1990 was chosen as the term *eHealth* was first introduced that year [49]. Updated searches were conducted in each database to include studies published by September 2021. The search strategy can be found in Multimedia Appendix 1. Medical Subject Heading indexing and free-text terms were used, and the search was adapted to each of the databases. In addition, relevant studies were identified via an ancestry approach—searching through the reference lists of the included papers and available systematic reviews.

Using the predefined criteria, all searches were conducted by the first author (AS) based on the abstract and title. The search results were imported into the EndNote bibliographic software (version 20; Clarivate Analytics). Duplicate records were removed, first automatically and then manually, and the remaining records were exported for screening. In total, 2 authors (AS and NH) screened all the records based on titles and abstracts and excluded those not fulfilling the inclusion criteria. Full texts were retrieved and then read for potentially eligible studies by 2 authors (AS and NH). Any disagreements were resolved through discussion and consultation with a third reviewer (KKB).

### Data Extraction

Data extraction forms were developed (RWA and AS) and used to create summary tables. Data were extracted independently by pairs of reviewers (AS+KKB, NH+MS, KS+RWA, and MMT+LSS), and quality was assured by a third reviewer (AS). The following data were extracted: author, year, country, setting, aim and outcomes, study design, control, sample size, type of sample (target or universal), participant characteristics, intervention description (pure or blended, mode of delivery, intervention components, duration of intervention, and incentives), adherence and attrition, and main study results including effect sizes.

### Study Quality Assessment

Risk of bias was assessed using the Cochrane Collaboration risk-of-bias tool for randomized trials (RoB 2) [50]. In the RoB 2 tool, bias is assessed via responses to signaling questions from 5 risk-of-bias domains: bias arising from the randomization process, bias because of deviations from the intended interventions, bias because of missing outcome data, bias in measurement of the outcome, and bias in selection of the reported results [50]. Signaling questions aim to inform the risk-of-bias assessment [50], and answering them enables the algorithm to produce the judgments, such as *high* risk of bias, *some concerns*, or *low* risk of bias. In total, 2 reviewer pairs (AS+KKB and AS+MMT) independently scored each study. Any discrepancies in grading were discussed and resolved through consensus.

### Effect Sizes

A meta-analysis was not considered feasible because of the high heterogeneity across the study outcomes and time points. Some studies reported changes in the health behavior measured, whereas others reported absolute values. Effect sizes were reported as various measures across the studies (Cohen *d*, *η*^2^, *η*^2^_p_, and Glass Δ). In cases in which the effect size was not reported and data were available, the Cohen *d* was calculated. Owing to the heterogeneity, the data were summarized narratively and visually (see Multimedia Appendix 2 [51-67] for the effect sizes of each study). Regarding interpretation, effect sizes were considered small if Cohen *d* and Glass Δ values were 0.20 and *η*^2^ and *η*^2^_p_ values were 0.01, medium if Cohen *d* and Glass Δ values were 0.50 and *η*^2^ and *η*^2^_p_ values were 0.06, and large if Cohen *d* and Glass Δ values were 0.80 and *η*^2^ and *η*^2^_p_ values were 0.14 [68].

### Mapping of BCTs

BCTs were mapped in accordance with the BCTTv1 by Michie et al [38], who argued that the potential benefits of having such a taxonomy include *accurate replication*, *faithful implementation*, *intervention development*, and *exploring mechanisms of action* [38]. The BCTTv1 includes 93 BCTs hierarchically structured into 16 groups containing a range of different BCTs, from *goal setting* and *problem solving* to *reward approximation*. Before mapping, 2 authors (AS and KS) participated in BCTTv1 training on the web [69]. Mapping was then performed independently by the same author pair (AS and KS) for all the included studies. Any discrepancies were resolved through consensus discussions. Information about the interventions was gathered using the available data found in the papers, supplementary materials, previously published protocols, or publications by the same author group.

## Results

### Selection of Studies

**Figure 1:**
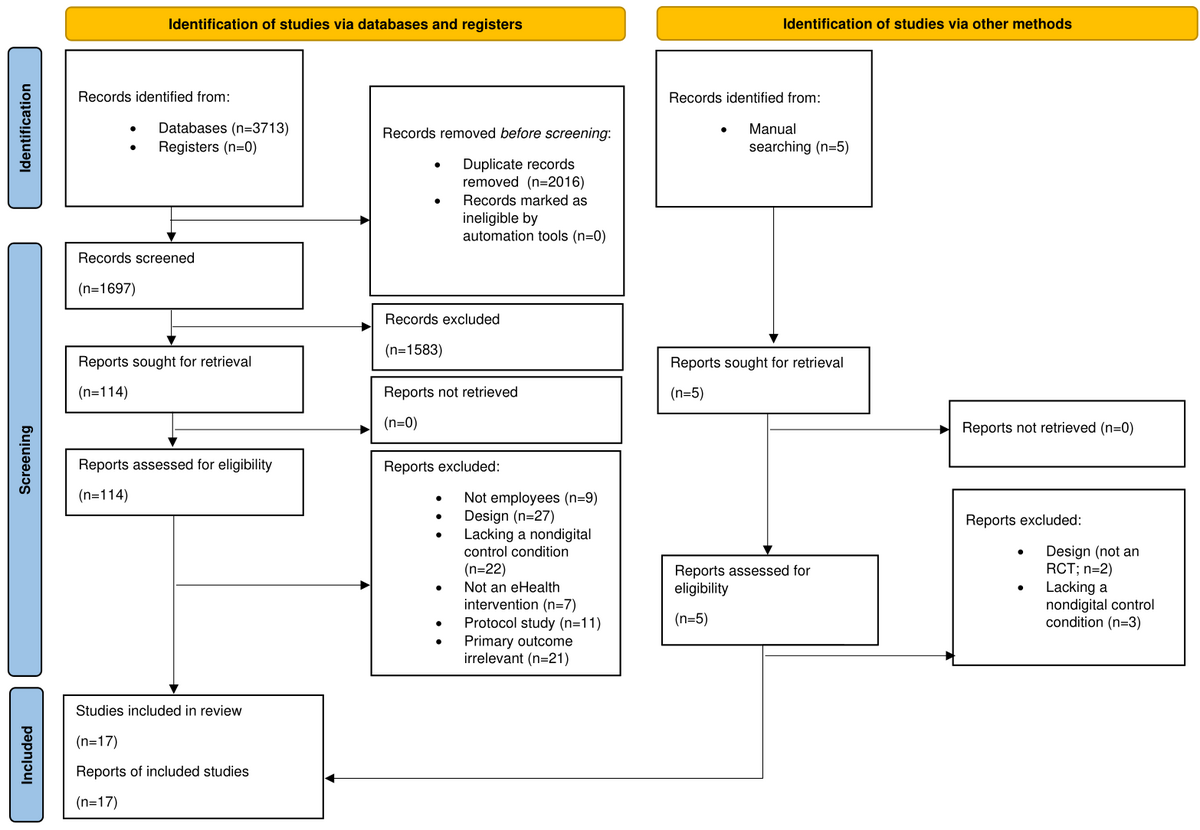
PRISMA (Preferred Reporting Items for Systematic Reviews and Meta-Analyses) [43] flow diagram of the search and study selection process. RCT: randomized controlled trial.

A PRISMA flowchart of the study selection process is presented in Figure 1. A total of 3718 records were identified via the initial searches, an updated search in September 2021, and the ancestry approach (n=3713, 99.87% through database searching and n=5, 0.13% through other sources). After deduplication, 1702 records were exported for screening, where 1583 (93%) publications were subsequently excluded. Full texts of potentially eligible studies were retrieved. Of the 119 papers, 17 (14.3%) met the inclusion criteria and were included in the review.

### Study Characteristics

A total of 17 studies published between January 1990 and September 2021 were included. The study characteristics are presented in Table 1. Of the 17 studies, 9 (53%) were conducted in the United States [51-59]; 2 (12%) were conducted in Germany [60,61]; 2 (12%) were conducted in Australia [62,63]; and 1 (6%) was conducted in the United Kingdom [64], Finland [65], Singapore [66], and the Netherlands [67] each. Workplace settings included hospitals and health care [55,57,58], academic institutions [51,59,62,64], insurance companies [65], human resources [53], IT companies [52], manufacturing facilities [55,60], and others [54,60,63,66,67].

Of the 17 studies, 7 (41%) compared an eHealth intervention targeting employees with a waitlist control [51,52,57,58,60,61,63], 5 (29%) compared it with a care-as-usual condition [54-56,59,66], and 5 (29%) used an active control condition [53,62,64,65,67]. A total of 65% (11/17) of the studies performed an intention-to-treat analysis [52,55,57-60,62,63,65-67], whereas 35% (6/17) completed a per-protocol analysis [51,53,54,61,64,66]. Attrition ranged from 0% to 37% and was either extracted from the text or calculated based on the available data. See Table 1 for details.

**Table 1:** Study design and attrition (N=17).

Study		RCT^a^ arms, N	Control condition	Follow-up (longest)	Incentives	Attrition
**Physical activity or sedentary behavior interventions**						
	Carr et al [51], 2013	2	Waitlist	12 weeks	Not specified	18.4%
	Irvine et al [56], 2011	2	Care as usual	1 month	Financial	7%
	Reijonsaari et al [65], 2012	2	Active control	12 months	Not specified	IG^b^: 33.5%; CG^c^: 30%
	Slootmaker et al [67], 2009	2	Active control	8 months	Not specified	IG: 19.6%; CG: 15.7%
	Poirier et al [57], 2016	2	Waitlist	6 weeks	Virtual rewards+activity tracker+gift card	18%
	Evans et al [64], 2012	2	Active control	5 days	No	6.5%
	Marshall et al [62], 2003	2	Active control	10 weeks	Not specified	22%
	Dadacyznski et al [61], 2017	2	Waitlist	6 weeks	Not specified	19%
	Finkelstein et al [66], 2016	4	Care as usual	12 months	During 6 months: financial and donations	19%
	Thorndike et al [58], 2014	2	Waitlist	6 weeks (phase 1)	Gift card lottery	4.8%
	Urda et al [59], 2016	2	Care as usual	2 weeks	No	0%
**Alcohol interventions**						
	Boß et al [60], 2017	3	Waitlist	6 months	Not specified	Unguided IG: 25%; guided IG: 26%; CG: 15%
	Doumas and Hannah [54], 2008	3	Care as usual	30 days	Financial or movie tickets	37%
**Multiple health behavior interventions**						
	Cook et al [53], 2007	2	Active control	3 months	Financial+lottery	Web group: 15%; CG: 13%
	Cook et al [52], 2015	2	Waitlist	3 months	Financial+lottery	10.07%
	Deitz et al [55], 2014	2	Care as usual	1 month	Financial+lottery	11%
	Oftedal et al [63], 2019	2	Waitlist	4 weeks	Gift card	15%

^a^RCT: randomized controlled trial.

^b^IG: intervention group.

^c^CG: control group.

### Sample Characteristics

The sample sizes ranged from 30 to 800 participants, and 4567 employees were recruited and randomized. More than half (10/17, 59%) of the studies recruited from a target population (eg, sedentary employees, risky drinkers, or young or older employees) [51,52,54-56,58-60,63,67], whereas 41% (7/17) of the studies included all employees [53,55,57,62,64-66]. A total of 76% (13/17) of the studies [51,53-55,57,58,60,63-67] had a higher proportion of female participants (≥50%), including a study that exclusively recruited female participants [59]. Highly educated participants were overrepresented in 71% (12/17) of the studies [51-53,55-60,62,66,67], meaning that more than half of the sample had a bachelor’s degree or other advanced degrees (see Multimedia Appendix 3 [51-67] for details).

### Target Behavior

A total of 65% (11/17) of the studies targeted primarily PA or sedentary behavior (SB) [51,56-59,61,62,64-67]. In total, 24% (4/17) of the studies focused on multiple health behaviors (ie, lifestyle change, PA promotion, healthy eating, and diet management) [52,53,55,63]. A total of 12% (2/17) of the studies focused on reduction in alcohol consumption [54,60]. We did not identify any studies that targeted nutrition or dietary practices and smoking exclusively that fulfilled the inclusion criteria. However, these health behaviors were identified and included in the studies that targeted multiple health behaviors.

### Intervention Characteristics

The intervention characteristics are summarized in Multimedia Appendix 4 [51-67]. The duration of the interventions varied greatly, ranging from 1 day (1 session) to 12 months. Follow-up length was also considerably different, with the shortest being 5 days and the longest being 12 months. A total of 88% (15/17) of the studies were delivered via websites, either exclusively or in combination with activity trackers, software, or email. In total, 41% (7/17) of the interventions used activity monitoring devices as part of the intervention. Only 6% (1/17) of the interventions were delivered via an app. Intervention intensity varied, but the most frequent delivery intensity was either daily or ad lib. Interventions were considered *pure* eHealth interventions when there was no human guidance involved and *blended* when a human component was a part of the intervention. A total of 82% (14/17) of the studies evaluated pure eHealth interventions, 6% (1/17) of the studies evaluated blended interventions, and 12% (2/17) of the studies evaluated both pure and blended interventions.

### Quality Assessment

**Figure 2:**
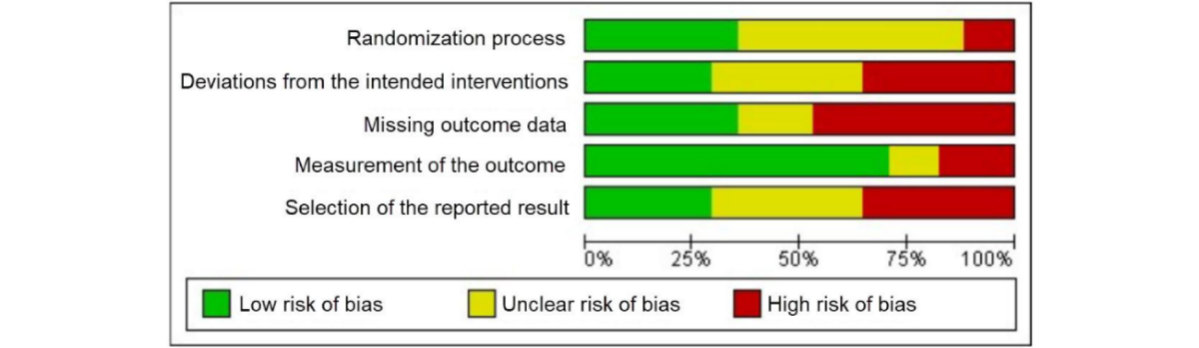
Risk of bias of the included studies.

Using the Cochrane RoB 2 tool, no studies were deemed to have a *low* risk of bias. In total, 35% (6/17) of the studies [57,58,60,64-65] had *some concerns* as they either failed to report efforts to avoid bias or did not report them in sufficient detail. A total of 65% (11/17) of the studies had a *high* *risk of bias* [51-56,69,61-63,67], implying considerable sources of bias that may have had a systematic impact on the results of the study. A summary of the risk-of-bias and quality assessment of the included studies is presented in Table 2 and visualized in Figure 2.

**Table 2:** Risk of bias of the included studies.

Study	Randomization process	Deviations from the intended interventions	Missing outcome data	Measurement of the outcome	Selection of the reported results
Carr et al [51], 2013	SC^a^	H^b^	H	L^c^	H
Irvine et al [56], 2011	H	L	H	L	H
Reijonsaari et al [65], 2012	L	SC	L	SC	L
Slootmaker et al [67], 2009	L	SC	H	SC	L
Poirier et al [57], 2016	SC	SC	L	L	L
Evans et al [64], 2012	L	L	L	L	SC
Marshall et al [62], 2003	L	SC	H	H	SC
Dadaczynski et al [61], 2017	H	H	H	H	SC
Finkelstein et al [66], 2016	SC	SC	L	L	SC
Thorndike et al [58], 2014	SC	SC	L	L	L
Urda et al [59], 2016	SC	H	H	L	SC
Boẞ et al [60], 2017	L	L	L	L	SC
Doumas and Hannah [54], 2008	SC	H	H	L	H
Cook et al [53], 2007	SC	H	SC	L	H
Cook et al [52], 2015	SC	H	SC	L	H
Deitz et al [55], 2014	SC	L	SC	L	H
Oftedal et al [63], 2019	L	L	H	H	L

^a^SC: some concerns.

^b^H: high risk of bias.

^c^L: low risk of bias.

### PA and SB Outcome Measures

#### Overview

A total of 65% (11/17) of the studies targeted either the PA or SBs of employees. The methodological quality was low to moderate (see Table 2 for details), with 45% (5/11) of these studies [57,58,64-66] having *some concerns* regarding the risk of bias and 55% (6/11) having a *high* risk of bias [51,56,59,61,62,67]. Interventions ranged from having just 2 BCTs pertaining to 1 group of BCTs (ie, *goals and planning*) [62] to some that contained 7 different BCTs belonging to 7 different groups (ie, *goals and planning*, *feedback and monitoring*, *social support*, *repetition and substitution*, *natural consequences, reward and threat*, and *antecedents*) [61]. All but 2 [59,64] of the interventions targeting PA and SB (9/11, 82%) were delivered via website, alone or in combination with activity trackers or pedometers, software, or email (see Table 1 for details). In total, 91% (10/11) of the studies [51,56-59,61,62,64,66,67] evaluated pure eHealth interventions not using any human support, guidance, or counseling, and only 9% (1/11) evaluated blended eHealth interventions combining telephone counseling with software, a web-based service, and accelerometers [65].

#### Studies With “Some Concerns” Regarding the Risk of Bias

A total of 45% (5/11) of the studies targeting PA or SB had *some concerns* regarding the risk of bias. Of these 5 studies, 2 (40%) reported no significant differences between the groups on all outcomes [58,65], 2 (40%) reported mixed results [64,66], and 1 (20%) reported significant between-group differences in all outcomes [57]. Post hoc analyses, no previously published protocol or analysis plan, lack of blinding, and lack of detailed reporting of randomization procedures led to the *some concerns* grading.

In total, 40% (2/5) of the studies in this group reported no significant differences. In the study by Reijonsaari et al [65], no significant effects were reported on PA and productivity-related outcomes. The intervention was delivered via an accelerometer, and Finnish insurance company employees were offered distance counseling. However, the frequency and duration of counseling were not reported in enough detail to ascertain the role that counseling may have played in the intervention. The follow-up period was 12 months, and the attrition rates were high at 32%. Thorndike et al [58] found that wearing an activity monitor with feedback and gamification did not have a significant impact on the PA of physicians in training at the 6-week follow-up.

In total, 40% (2/5) of the studies reported mixed findings. The study by Evans et al [64] had low attrition (Table 1) and mostly significant findings with small to large effect sizes. The authors investigated the effectiveness of a desktop prompting software on reducing long uninterrupted sedentary periods and total sedentary time in mostly female (73%) university employees. The intervention was delivered via a desktop prompting software for 5 days. At follow-up, there was no significant difference between the groups in total time spent sitting. However, the intervention group had a significantly reduced number and duration of prolonged sitting events (uninterrupted sitting for >30 minutes). Finkelstein et al [66] compared the effectiveness of activity trackers with or without incentives for increasing PA. At the 6-month follow-up, there were no significant differences in moderate to vigorous PA or steps between the Fitbit-only and control groups. At the 12-month follow-up, participants in the Fitbit-only group had an increase in moderate to vigorous PA in comparison with the control group, although 90% of the participants had stopped wearing the activity tracker by then.

In total, 20% (1/5) of the studies reported significant between-group differences in PA (steps per day) at follow-up compared with the controls. Participants in a 6-week combined activity tracker and internet-based adaptive program [57] designed to increase light PA reported a significant increase in steps (970 steps per day) in comparison with a control group. The intervention was tailored and offered by dynamically adjusting the goals to the participants.

#### Studies With “High” Risk of Bias

A total of 35% (6/17) of the studies targeting PA or SB had a *high* risk of bias. Of these 6 studies, 1 (17%) found significant between-group differences in all outcomes [56], 3 (50%) [51,59,61] reported mixed findings, and 2 (33%) found no significant between-group differences in any PA or SB outcomes [62,67] (see Multimedia Appendix 2 for details).

In total, 17% (1/6) of the studies in this group found significant between-group effects in all outcomes. Irvine et al [56] evaluated the effectiveness of an eHealth intervention for increasing the PA of sedentary employees in a large manufacturing plant, mostly men (58%), with an average BMI of 29.5. The intervention group had greater effects on all outcomes, with effect sizes ranging from small to large. Participants reported a significantly greater frequency of PA and a significant increase in minutes per day of activity but also a significant increase in knowledge, attitude, and behavioral intention regarding PA. The intervention group also reported increased motivation to be physically active compared with the control group, as well as an increase in behavioral self-efficacy in relation to PA (ie, confidence that they could maintain healthy levels of PA) and reduced perceived barriers to PA.

In total, 50% (3/6) of the studies in this group reported mixed findings. Carr et al [51] compared the effectiveness of an eHealth intervention in reducing SBs in mostly female (90%) sedentary and overweight university employees with a control group. At the 12-week follow-up, participants in the intervention group showed significantly reduced daily sedentary time compared with the control group after adjusting for baseline sedentary time and monitoring time wear. No significant differences between the groups were found in the other outcomes. Dadaczynski et al [61] compared the effectiveness of a pedometer-based and gamified web-based intervention aimed at promoting low levels of PA (walking) in mostly male (65%) employees of German automobile manufacturers with a control group. At the 6-week follow-up, there was a significant increase in walking (125 min per week) in the intervention group but no differences for moderate and high PA. Urda et al [59] evaluated the effectiveness of an alert to get up once per hour on workplace sitting and perceived wellness in middle-aged women. After 2 weeks, there was a significant difference between the groups in average sitting time but not in other outcomes.

In total, 33% (2/6) of the studies in this group reported no significant between-group effects on any of the PA or SB outcomes [62,67]. Marshall et al [62] compared the effectiveness of an interactive website and email program to increase PA at an Australian university (71% of the participants had postsecondary education) with a print version of the same program. At the 10-week follow-up, there were no significant differences in reported PA between the groups. Slootmaker et al [67] investigated the effectiveness and feasibility of an eHealth intervention for increasing PA in mostly female (60%) Dutch office workers aged between 20 and 40 years. The intervention was delivered via a website with tailored PA advice, software, and an activity monitoring device. The authors found no significant differences in any of the outcomes in the total study population at either the 3- or 8-month follow-ups.

### Alcohol Consumption

A total of 12% (2/17) of the included studies targeted employee alcohol consumption. One study in this group was assessed as having *some concerns* regarding the risk of bias [60], and the other was assessed as having a *high* risk of bias [54]. The interventions differed greatly in terms of the number of BCTs used. Boẞ et al [60] reported 15 different BCTs (from goals and planning to self-belief spanning 9 different groups), whereas Doumas and Hannah [54] reported only 4 different BCTs belonging to 3 groups (feedback and monitoring, natural consequences, and comparison of behavior). Both studies were 3-arm RCTs comparing 2 intervention groups (guided and unguided) with either a waitlist [60] or a care-as-usual control group [54]. Pure (unguided) interventions in both studies were delivered via a website, but in the study by Boẞ et al [60], the web-based intervention consisted of 5 different modules that participants were advised to complete over 5 weeks, whereas in the study by Doumas and Hannah [54], participants were given access to a brief web-based program (1 session) with personalized normative feedback. Regarding the blended (guided) interventions, in the study by Boẞ et al [60], participants were assigned an “eCoach” throughout the study with the aim of encouraging adherence to the intervention and providing optional feedback to the participants, whereas the participants in the study by Doumas and Hannah [54] received a 15-minute motivational interview with a trained counselor.

The study by Boẞ et al [60] had *some concerns* regarding the risk of bias. The authors compared the efficacy of a web-based alcohol intervention with and without guidance with a waitlist control in middle-aged (mean 47 years), mostly well-educated (67.5%) risky drinkers in Germany. The intervention included different modules with information, exercises, multimedia files, and personalized normative feedback. The authors found no significant differences between the unguided and guided web intervention groups but found a significant reduction in mean weekly alcohol consumption for the web groups when compared with the control group at both the 6-week and 6-month follow-ups, with moderate effect sizes (see Multimedia Appendix 2 for details).

The study by Doumas and Hannah [54] was assessed as having a *high* risk of bias. The authors compared the efficacy of an alcohol prevention web-based intervention with personalized feedback with the same web-based intervention plus a 15-minute motivational interview session and a control condition in young, mostly female (73%) employees in the United States. Attrition was high at 37%. Participants in the intervention groups reported a significant decrease in the quantity of weekend drinking, frequency of drinking to intoxication, and peak consumption at the 30-day follow-up. There were no significant differences in any of the outcomes between the blended and pure intervention groups.

### Smoking or Tobacco Use

Our search did not identify any RCTs targeting employee tobacco use exclusively that fulfilled the inclusion criteria. However, some of the studies targeting multiple health behaviors (2/17, 12%) also included smoking cessation or reduction as one of the outcomes [52,55]. Regarding the impact of eHealth interventions on tobacco use, 6% (1/17) of the studies did not include enough participants to perform the analyses on this outcome [52], whereas the study by Deitz et al [55] found small intervention effects on smoking status among the few included smokers in the study. Both the aforementioned studies were assessed as having a high risk of bias.

### Diet

Our search did not identify any RCTs targeting employee nutritional patterns or diet exclusively that fulfilled the inclusion criteria. However, diet-related outcomes were found in the studies evaluating eHealth interventions targeting multiple health behaviors (4/17, 24%) [52,53,55,63]. All the aforementioned studies were judged as having a high risk of bias because of either not reporting measures taken to avoid bias or being judged as having serious sources of bias that could affect the study results. Oftedal et al [63] reported a positive effect on diet quality in participants in the intervention group relative to the control group. Cook et al [53] reported a significant change in dietary stage of change for participants in the web-based program (*P*=.01). Deitz et al [55] found significant differences between the intervention and control groups in 3 out of 4 dietary outcomes (dietary attitudes, dietary intentions, and dietary self-efficacy), whereas Cook et al [52] found significant improvement in diet behavior change self-efficacy and planning healthy eating but not in other dietary outcomes.

### Interventions Targeting Multiple Health Behaviors

In total, 24% (4/17) of the studies targeted multiple health behaviors [52,53,55,63]. All studies (4/4, 100%) were assessed as having a *high* risk of bias. Interventions varied in terms of the number of BCTs used, from just 1 [55] to 15 [53,63]. A total of 75% (3/4) of these interventions were delivered [52,53,55] via website, and 25% (1/4) were delivered via an app [63]. Studies targeted different health behaviors. All of them included diet and PA as health behaviors; 50% (2/4) in addition included tobacco use [52,55]; and 25% (1/4) combined diet, PA, and sleep quality [63].

Cook et al [53] evaluated the effectiveness of a web-based multimedia health promotion program aimed at improving dietary practices, reducing stress, and increasing PA in mainly female (72%) and highly educated (94%) employees. The intervention group showed significant improvement in attitudes toward a healthy diet and dietary stage of change. However, the magnitude of the effect was small. Another study by Cook et al [52] aimed to evaluate the effectiveness of a web-based health promotion program, HealthyPast50, on multiple health behaviors in older, mainly male (69%), and highly educated employees (65%). The intervention group significantly improved on diet behavior change self-efficacy and planning healthy eating but not on other dietary measures. The intervention group also improved significantly on “mild” exercise (exercise requiring minimal effort, such as yoga, fishing, and bowling [71]). The magnitude of the effect varied greatly (see Multimedia Appendix 2 for details). Deitz et al [55] evaluated the effectiveness of a web-based cardiovascular health promotion program targeting PA, diet, and smoking, among other outcomes, in mostly female (86%) and highly educated (87%) hospital employees. At follow-up, the intervention group significantly improved on dietary attitudes, intentions, and self-efficacy, albeit with small effect sizes. The intervention group also improved on exercise self-efficacy and exercise behaviors (especially strenuous PA). The magnitude of the effect was small across all PA outcomes except for strenuous PA, where the intervention produced moderate effects. Finally, Oftedal et al [63] evaluated the preliminary effectiveness of a mobile health intervention compared with a waitlist control in shift workers in Australia. Outcomes included PA and diet quality. The authors found significant improvements in diet quality in the intervention group with a large effect size but not in other outcomes.

### BCTs Used in the Studies

BCTs were mapped using the BCTTv1 by Michie et al [38]. A complete overview of the identified BCTs is available in Table 3. The most commonly used BCTs were in the *feedback and monitoring* group and were identified in 76% (13/17) of the studies, followed by *goals and planning* in 59% (10/17) of the studies and *antecedents*—mainly *adding objects to the environment* in 53% (9/17) of the studies. *Social support* and *natural consequences* (mostly *information about the health consequences*) were also quite commonly used components in the interventions and were found in 41% (7/17) of the studies, respectively. *Associations* were found in 24% (4/17) of the studies. Shaping knowledge, *repetition and substitution*, *regulation*, and *comparison* *of behavior* were each used in 18% (3/17) of the studies. BCTs belonging to the groups *comparison of outcomes* and *reward and threat* were used in 12% (2/17) of the studies, whereas BCTs adhering to the *self-belief* group were used in only 6% (1/17) of the studies. Of the 16 groups of BCTs, 3 (19%; *identity*, *scheduled consequences*, and *covert learning*) were not identified in any of the studies. The interventions targeting alcohol consumption included BCTs for *self-belief* and *comparison of behavior* (*social comparison*), whereas interventions for other health behaviors did not include these groups of BCTs.

**Table 3:** Overview of the behavior change techniques (BCTs) across the studies (N=17).

Grouping and BCTs		Studies, n (%)	References
**Goals and planning**		10 (59)	
	Goal setting (behavior)	9 (53)	Carr et al [51] Cook et al [53] Irvine et al [56] Poirier et al [57] Boß et al [60] Dadaczynski et al [61] Marshall et al [60] Oftedal et al [63] Reijonsaari et al [65]
	Problem-solving	5 (29)	Cook et al [53] Irvine et al [56] Boß et al [60] Oftedal et al [63] Slootmaker et al [67]
	Goal setting (outcome)	2 (12)	Oftedal et al [63] Slootmaker et al [67]
	Action planning	4 (24)	Irvine et al [56] Marshall et al [62] Oftedal et al [63]
	Review behavior goal(s)	1 (6)	Oftedal et al [63]
**Feedback and monitoring**		13 (76)	
	Monitoring of behavior by others without feedback	2 (12)	Reijonsaari et al [65] Finkelstein et al [66]
	Feedback on behavior	9 (53)	Cook et al [52,53] Doumas and Hannah [54] Irvine et al [56] Poirier et al [57] Thorndike et al [58] Dadaczynski et al [61] Oftedal et al [63] Finkelstein et al [66] Slootmaker et al [67]
	Self-monitoring of behavior	6 (35)	Carr et al [51] Cook et al [53] Irvine et al [56] Thorndike et al [58] Oftedal et al [63] Reijonsaari et al [65]
	Self-monitoring of outcome of behavior	1 (6)	Oftedal et al [63]
	Monitoring of outcomes(s) of behavior without feedback	2 (12)	Cook et al [53] Reijonsaari et al [65]
	Biofeedback	2 (12)	Carr et al [51] Doumas and Hannah [54]
	Feedback on outcomes(s) of behavior	6 (35)	Carr et al [51] Cook et al [53] Poirier et al [57] Thorndike et al [58] Oftedal et al [63] Slootmaker et al [67]
**Social support**		7 (41)	
	Social support (unspecified)	6 (35)	Cook et al [53] Irvine et al [56] Boß et al [60] Dadaczynski et al [61] Oftedal et al [63] Slootmaket et al [67]
	Social support (practical)	1 (6)	Carr et al [51]
	Social support (emotional)	1 (6)	Carr et al [51]
**Shaping knowledge**		3 (18)	
	Instruction on how to perform the behavior	3 (18)	Cook et al [53] Urda et al [59] Boß et al [60]
	Information about antecedents	1 (6)	Cook et al [53]
	Reattribution	1 (6)	Boß et al [60]
**Natural consequences**			
	Information about health consequences	7 (41)	Cook et al [52] Doumas and Hannah [54] Deitz et al [55] Urda et al [59] Dadaczynski et al [61] Oftedal et al [63] Evans et al [64]
**Comparison of behavior**			
	Social comparison	3 (18)	Doumas and Hannah [54] Boß et al [60] Dadaczynski et al [61]
**Associations**			
	Prompts or cues	4 (24)	Carr et al [51] Urda et al [59] Oftedal et al [63] Evans et al [64]
**Repetition and substitution**		3 (18)	
	Behavioral practice or rehearsal	1 (6)	Cook et al [53]
	Behavioral substitution	2 (12)	Boß et al [60] Dadaczynski et al [61]
**Comparison of outcomes**		2 (12)	
	Credible source	1 (6)	Cook et al [53]
	Pros and cons	1 (6)	Boß et al [60]
**Reward and threat**		3 (18)	
	Nonspecific reward	1 (6)	Poirier et al [57]
	Social reward	2 (12)	Poirier et al [57] Dadaczynski et al [61]
**Regulation**		3 (18)	
	Reduce negative emotions	2 (12)	Boß et al [60] Oftedal et al 65]
	Conserving mental resources	1 (6)	Cook et al [53]
**Antecedents**		10 (59)	
	Restructuring the physical environment	2 (12)	Carr et al [51] Oftedal et al [63]
	Avoidance or reducing exposure to cues for the behavior	1 (6)	Cook et al [53]
	Adding objects to the environment	9 (53)	Carr et al [51] Cook et al [53] Poirier et al [57] Thorndike et al [58] Urda et al [59] Dadaczynski et al [61] Reijonsaari et al [65] Finkelstein et al [66] Slootmaker et al [67]
	Body changes	1 (6)	Cook et al [53]
**Self-belief**		2 (12)	
	Verbal persuasion about capability	1 (6)	Boß et al [60]
	Mental rehearsal of successful performance	1 (6)	Boß et al [60]

### Intervention Effects Across the Included Studies

#### Unequivocal Significant Findings

**Figure 3:**
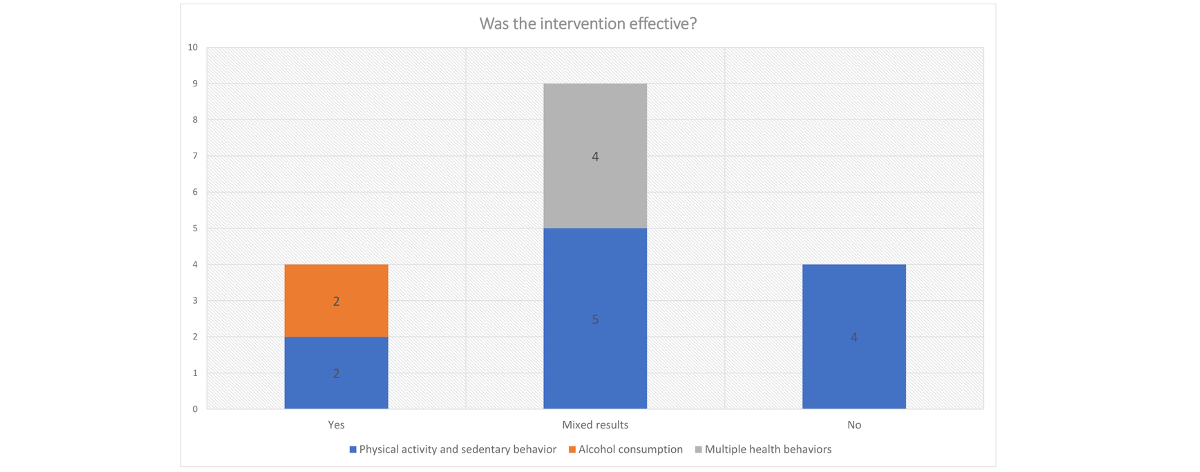
Effectiveness of interventions across the included studies.

Of the 17 studies, 4 (24%) had positive significant findings for all primary outcomes, with varying magnitudes of the effects measured (see Multimedia Appendix 2 for effect sizes and Figure 3 for an overview of effectiveness). In total, 50% (2/4) of the studies that demonstrated statistically significant effects targeted employee alcohol consumption—one evaluated a 5-week web-based intervention incorporating 15 different BCTs and reported small intervention effects on primary outcomes (effects were somewhat greater for a blended intervention compared with the pure intervention) [60], and the other evaluated a session of a web-based personalized feedback that used 4 BCTs [54] and reported small to large effects on primary outcomes. The other 50% (2/4) of the studies with significant findings targeted PA: a 6-week web-based adaptive walking program with an activity tracker [57] reported a moderate effect on daily steps, and a 28-day Get Moving, website-only PA intervention [56] reported a large effect on self-reported PA, both using 6 different BCTs.

#### Mixed and Nonsignificant Findings

A total of 53% (9/17) of the included studies reported mixed results; of these 9 studies, 5 (56%) targeted PA [51,59,61,64,66], whereas 4 (44%) targeted multiple health behaviors [52,53,55,63]. However, in 67% (6/9) of these studies, most of the outcomes were not significantly different between the groups [51-53,59,63,66]. These studies were mostly delivered via websites (either exclusively or accompanied by an activity tracker or pedometer), with the exception of 22% (2/9) that were delivered via computer prompts [57,59] and 11% (1/9) that were delivered using an app [63]. The studies varied in delivery intensity from 5-day [64] to 3-month interventions [51-53] and used from 2 to 15 BCTs.

Of the 17 included studies, 4 (24%) showed no significant differences between groups in any of the outcomes measured [58,62,65,67]. Regarding the use of BCTs, mode of delivery, or intensity of the interventions, these studies were not considerably different from the studies with positive or mixed findings. All of them (4/4, 100%) targeted PA [58,62,65,67], and the BCTs ranged from 2 [62] to 6 [67].

Only 6% (1/17) of the studies evaluated an intervention that targeted organizational outcomes in addition to a health behavior [65].

## Discussion

### Principal Findings

This systematic review provides a comprehensive overview of the effectiveness of eHealth interventions aimed at improving employee health behaviors and maps the BCTs used in these interventions to promote behavior change. A total of 17 RCTs published between 2003 and 2019 targeting employee PA or SB, dietary practices, alcohol consumption, and smoking were included. Significant moderate to large effects were reported by more than half (10/17, 59%) of the studies on at least one of the primary outcomes; however, more than two-thirds (13/17, 76%) of the studies also reported mixed and nonsignificant results with small effects on most of the evaluated outcomes. Owing to the large heterogeneity of contexts, modes of delivery of eHealth technologies, contents of the interventions, BCTs used, and treatment and follow-up periods, combined with poor methodological quality, it was not possible to draw definitive conclusions on the effectiveness of different types of interventions, including BCTs.

The included studies covered a broad range of eHealth interventions across various types of workplaces and outcomes. The most frequent mode of delivery for eHealth interventions was websites alone or combined with activity trackers, software, or email, whereas only 6% (1/17) of the included studies used a mobile app. Although they used the same mode of delivery, the contents and design of the interventions varied greatly, which added to the heterogeneity and made comparisons in effectiveness between interventions challenging.

Some health behaviors were relatively more frequently targeted in the sample (ie, PA), whereas others were less targeted (tobacco use). The latter finding may be explained by the decline in tobacco use over the last few years worldwide but also by a lower percentage of tobacco users in high-income countries than in low-income countries [71].

The extensive focus on activity and SBs in the studies included in this review could be explained by several factors. As this review focused on employees, SBs may be particularly relevant when it comes to office workers. Widespread physical inactivity [72,73] with a plethora of consequences concerns the health of the individual but also of organizations through effects on work productivity and sickness absence. In addition, previous studies have reported that the effect of PA depends on whether PA is performed at work (occupational PA) or after work (leisure-time PA) [75-76]. This is known as the *physical activity paradox* and describes the health benefits of leisure-time PA versus the sometimes detrimental health effects of occupational PA [77]. In other words, increasing PA levels among sedentary employees is beneficial. However, this finding differs from the findings of a review of eHealth interventions targeting health behaviors and obesity among young adults in general [32] in which alcohol and smoking studies dominated over studies on other health behaviors. The sample selection from the workplace context in this review, as well as age and different inclusion criteria, may have contributed to these differences.

The included studies were exclusively conducted in high-income countries, with more female participants and participants with high educational attainment than in the general population, thus limiting the generalizability of the results beyond the contexts of these studies. Henrich et al [78] have previously advised against assuming that results found in White, educated, industrialized, rich, and democratic populations are broadly representative. Furthermore, previous research has shown that individuals with higher educational attainment are more likely to have higher health literacy than those with lower education [79-81]. Thus, it might be challenging to achieve large intervention effects for preventive interventions among groups of people with already high health literacy, and those included in some of these studies may also have a lower risk of developing NCDs [82]. Accordingly, recruiting healthy participants, although some had sedentary jobs, might have masked the potential beneficial effects of the interventions in question.

The methodological quality of the included studies was low to moderate, with two-thirds (11/17, 65%) of the studies having a high risk of bias. Most studies (13/17, 76%) reported high attrition rates. With high attrition rates, many of the benefits of randomization are attenuated, which may lead to bias [47]. This may compromise subsequent inferences about the effects of treatment. Attrition also lowers power and increases the probability of committing type-II errors. The studies reporting the lowest attrition rates were those by Urda et al [59] with 0% attrition and by Thorndike et al [58], which reported attrition of <5%. A reason for the low attrition rates in these studies might be the relatively low invasiveness of the interventions evaluated in the studies (computer prompts or wearing the activity tracker only). However, the study by Doumas and Hannah [54] reported the highest attrition rates of all the included studies (37%) despite the fact that the alcohol prevention intervention was relatively short for both intervention groups (a 1-session web-based personalized feedback in one group plus a 15-minute motivational interview in the other group). However, high attrition rates are not unusual in web-based alcohol prevention interventions [83,84]. The reasons for dropping out of alcohol prevention interventions vary and may be associated with the stigma of receiving alcohol prevention interventions, not recognizing own alcohol use as problematic [85], threats to self-image [86], or being a heavy drinker [87]. Previous research has also found that poor methodological quality increases the average effect size almost 3-fold for studies of low quality compared with those of good quality [5].

Furthermore, the eHealth interventions were heterogeneous in content, outcomes, delivery mode, context, and BCTs used. All these aspects create complexity that affected the ability to draw any definitive conclusions on what drives the effect of the intervention and which BCTs are particularly effective for which outcome. However, the most used BCTs across the studies belonged to the *feedback and monitoring* group, especially in the case of interventions targeting PA and SB, and the BCT *goal setting* was the most widely used technique in this particular group. This is in line with previous research that found *goal setting* as one of the most used techniques among digital interventions targeting SB in clinical [88] and general populations [89-91], whereas *social support* was the most used BCT when engaging older populations in PA. BCTs targeting goal setting, feedback, and self-monitoring are also common in eHealth interventions delivered via apps to improve diet, PA, and SB [92]. *Adding objects to the environment* was the most common BCT belonging to the *antecedents* group and was coded mostly in connection with providing participants with various activity trackers. Regarding the previous research on BCTs used in interventions targeting alcohol consumption, *behaviour substitution, problem solving*, and *goal setting* were associated with greater alcohol reduction in the study by Garnett et al [93], whereas *commitment, social comparison, feedback,* and *review of goals* [94] had a greater impact on alcohol reduction. Studies targeting alcohol consumption in this review used BCTs to considerably different degrees (4 BCTs in the study by Doumas and Hannah [54] vs 15 BCTs in the study by Boß et al [60]), whereas both reported a significant reduction in alcohol consumption.

Overall, based on the results of this review, it seems that, among those interventions that reported significant results and impact, over half (8/13, 62%) targeted only 1 health behavior and the reported effects were mostly short-term except for the study by Boß et al [60], which reported significant effects also after 6 months. Regarding the effect sizes, incomplete reporting of the data prevented effect size calculations in some cases. However, there was a high variation in effect sizes among the interventions in general and problems with methodological quality, among them high attrition and low power, which could have affected the results and increased the chance of type-II errors.

### Strengths and Limitations

The study followed the PRISMA guidelines for reporting systematic reviews [43]. The strengths of this study include the extensive use of independent pairs of researchers performing screening, data extraction, BCT mapping, and quality assessments as well as the use of the RoB 2 tool. To limit the heterogeneity of the studies, a crucial eligibility criterion was randomization at the individual unit level to lower the design heterogeneity and bias that might result from randomization at different levels. Moreover, we included only studies that compared eHealth interventions with a nondigital control group as we aimed to evaluate the effect of eHealth in comparison with nondigital interventions or no treatment and not the effectiveness of different delivery modes. In addition, the BCTs were mapped according to a reliable method for identifying and interpreting the BCTs used in eHealth interventions, which will hopefully facilitate replication and broaden the knowledge on the interventions used in these studies.

However, these results should be interpreted in light of some limitations. Despite the rigorous inclusion criteria, heterogeneity across the studies was still extensive, which prevented the drawing of definitive conclusions regarding the effectiveness of the interventions and BCTs used, as well as a meta-analysis. In addition, a high proportion of studies that were of poor methodological quality (11/17, 65%) limits the reliability of the findings. Another possible limitation is the small number of included studies targeting alcohol use, which may be due to the strict inclusion criteria. The mapping of BCTs proved to be challenging in some cases because of the vague descriptions of the interventions and lack of transparency in reporting the intervention content, which may have affected the validity of the mapping.

### Implications for Future Research

Future studies should aim for more transparency in reporting BCTs and to harness the full potential of RCT designs to elevate the methodological quality and reduce heterogeneity. New methods are needed to address these issues, such as a megastudy design in which different interventions are evaluated in the same population over the same period on the same outcomes, which may solve some of the challenges [95]. In addition, targeting healthy workers with high health literacy and high educational attainment might mask the potentially beneficial effects of health interventions and decrease generalizability. Therefore, further research and development of interventions targeting populations of lower socioeconomic status and lower health literacy might be beneficial.

### Conclusions

There is evidence from this review suggesting that, although eHealth interventions have potential, there are still many unanswered questions regarding their effectiveness and what drives the mechanisms behind these effects. Low methodological quality, high heterogeneity, characteristics of the included samples, and often high attrition rates challenge the investigation on the effectiveness of the interventions and the making of sound inferences about the effect sizes and significance of the results. Therefore, the effectiveness is still unclear. eHealth interventions have yet to deliver on their promise, and more high-quality studies are needed for them to do so.

## Appendix

Multimedia Appendix 1 Example of the search strategy.

Multimedia Appendix 2 Study outcomes and effect sizes.

Multimedia Appendix 3 Sample characteristics and study settings.

Multimedia Appendix 4 Intervention characteristics and outcome measurement.

## Abbreviations

BCT: behavior change technique
BCTTv1: Behavior Change Technique Taxonomy version 1
NCD: noncommunicable disease
PA: physical activity
PRISMA: Preferred Reporting Items for Systematic Reviews and Meta-Analyses
RCT: randomized controlled trial
RoB 2: risk-of-bias tool for randomized trials
SB: sedentary behavior
WHO: World Health Organization
WHPP: workplace health promotion program
