# Supplementary material for: Effectiveness of eHealth Interventions Targeting Employee Health Behaviors: Systematic Review
Source: J Med Internet Res. 2023 Apr 20;25:e38307. doi: 10.2196/38307 (PMC10160931; doi:10.2196/38307)
Supplement: Multimedia Appendix 1 [file jmir_v25i1e38307_app1.docx]

Multimedia Appendix 1. Example of the search strategy.

| Population | Intervention | Comparator | Outcome | Restricted by | Comment |
| --- | --- | --- | --- | --- | --- |
| employ* OR work* OR OR workplace [MeSH Terms] OR worksite | eHealth OR digital OR “computer-delivered” OR “web-based” OR app OR smartphone OR telemedicine [MeSH Terms] OR online OR “internet-delivered” | “care-as-usual” OR “control” OR “no treatment” OR “waitlist” OR “wait-list” OR “treatment-as-usual” OR paper OR “active control” | alcohol OR drinking OR "alcohol consumption" OR "risky drinking" OR "alcohol dependency" OR “hazardous drinking” | RCT in PubMed  ----------------  Trials in Cochrane | PubMed 94 results  Cochrane Central 18)  Embase 31  CINHAL 33  PsychINFO 538 |
|  |  |  | Smoke [MeSH Terms] OR tobacco [MeSH Terms] OR “smoking cessation” OR “cigarette smoking” |  | PubMed 77 results  Cochrane Central 29  Embase 10  CINHAL 28  PsychINFO 301 |
|  |  |  | nutrition OR diet [MeSH Terms] OR “healthy eating” |  | PubMed 172 results  Cochrane Central 3  Embase 50  CINHAL 57  PsychINFO 348 |
|  |  |  | “physical activity” OR “physical training” OR “sedentary behaviour” OR exercise [MeSH Terms] OR fitness OR sitting OR “prolonged sitting” |  | PubMed 299 results  Cochrane Central 49  Embase 60  CINHAL 113  PsychINFO 446 |
|  |  |  | obesity [MeSH Terms] OR obes* OR overweight OR high BMI OR “weight management” |  | PubMed 105 results  Cochrane Central 25  Embase 31  CINHAL 39  PsychINFO 373 |
|  |  |  | «sick leave» [MeSH Terms] OR «sickness absence” OR absent* |  | PubMed 57 results  Cochrane Central 17  Embase 16  CINHAL 30  PsychINFO 100 |
|  |  |  | (("Health behaviours") OR ("lifestyle behaviours")) OR ("lifestyle factors") |  | PubMed 17 results  Cochrane Central 19  Embase 6  CINHAL 62  PsychINFO 65 |

Example of the search strategy for PubMed

Records identified by search in different databases

| PubMed | 821 |
| --- | --- |
| Cochrane CENTRAL | 160 |
| Embase | 204 |
| CINAHL | 362 |
| PsychINFO | 2171 |
| Total | 3718 |
