# Supplementary material for: Effectiveness of eHealth Interventions Targeting Employee Health Behaviors: Systematic Review
Source: J Med Internet Res. 2023 Apr 20;25:e38307. doi: 10.2196/38307 (PMC10160931; doi:10.2196/38307)
Supplement: Multimedia Appendix 2 [file jmir_v25i1e38307_app2.docx]

Multimedia Appendix 2. Study outcomes and effect sizes.

| **Study** | **RoB** | **Between group differences (IG vs CG) for outcome at post-test - effect size** | | ***P value*** |
| --- | --- | --- | --- | --- |
| ***Physical activity or sedentary behaviour interventions*** | | | | |
| Carr et al 2013 [51] | High | **Primary outcome(s):** |  |  |
|  |  | Minutes sedentary  (-58.7 min/day reduction in IG) | d= - 0.82 | **.01** |
|  |  | % of time sedentary | d= - 0.45 | .06 |
|  |  | minutes light PA | d=0.12 | .64 |
|  |  | % of time light PA | d=0.30 | .16 |
|  |  | minutes moderate PA | d=0.22 | .13 |
|  |  | % of time moderate PA | d=0.25 | .16 |
|  |  | minutes vigorous PA | d=0.45 | .33 |
|  |  | % of time vigorous PA | d=0.43 | .25 |
|  |  | **Secondary outcome(s):** |  |  |
|  |  | Weight (lbs) | d= - 0.24 | .58 |
|  |  | BMI | d= - 0.31 | .76 |
|  |  | Waist circumference (cm) | d= - 0.21 | .03 |
| Irvine et al 2011 [56] | High | **Primary outcome(s):** |  |  |
|  |  | Current Exercise Status Scale (CESS) | η_p_^2^=0.26 | **<.001** |
|  |  | PA: minutes per day activity | η_p_^2^=0.22 | **<.001** |
|  |  | **Secondary outcome(s):** |  |  |
|  |  | Attitudes towards PA | η_p_^2^=0.15 | **<.001** |
|  |  | Perceived knowledge of PA | η_p_^2^=0.21 | **<.001** |
|  |  | Behavioral self-efficacy | η_p_^2^=0.06 | **.001** |
|  |  | Behavioral intention | η_p_^2^=0.15 | **<.001** |
|  |  | Barriers to PA | η_p_^2^=0.16 | **<.001** |
|  |  | Motivation to be physically active | η_p_^2^=0.08 | **<.001** |
| Reijonsaari et al 2012 [65] | Some Concerns | **Primary outcome(s):** |  |  |
|  |  | Physical activity (modified IPAQ, MET min/week) | d=-0.17 | *P -* not significant, values NA |
|  |  | body weight | d=-0.07 |  |
|  |  | waist circumference | d=-0.1 |  |
|  |  | body fat percentage | d=0.12 |  |
| Slootmaker et al 2009 [67] | High | **Primary outcome(s):** |  |  |
|  |  | PA MET minutes per week spent on: | No significant intervention effects on PA levels or any of the other outcomes in the total study population.  Subgroup analyses: Relative lowering of light-intensity PA (2-4 METs) among higher-educated participants post intervention. Not sustained at 8 months. | |
|  |  | Light-intensity PA | β  3 months: -84  8 months: -18 | (95% CI)  (-290.9;123.3)  (-220.6;185.1) |
|  |  | Moderate intensity PA | β  3 months: -22  8 months: 97 | (95% CI)  (-96;53)  (-47;241) |
|  |  | Vigorous intensity PA | β  3 months: -4  8 months: -17 | (95% CI)  (-71;63)  (-97;62) |
|  |  | Time spent sedentary | β  3 months: 101  8 months: -174 | (95% CI)  (-338; 540)  (-721; 374) |
|  |  | **Secondary outcomes:** |  |  |
|  |  | Determinants of PA | No sig. intervention effect. | |
|  |  | Aerobic Fitness test - Chester Step Test | No sig. intervention effect. | |
|  |  | Body composition (Body weight, BMI, Waist and hip circumference, Thickness of skin, % body fat) | No sig. intervention effect in total study population. Subgroup analyses: Decrease in body weight (-1.6kg) among low-educated IG participants compared to CG at post intervention and 8-month FU | |
| Poirier et al 2016 [57] | Some Concerns | **Primary outcome(s):** |  |  |
|  |  | Change in steps per day from baseline to follow-up for all | d=0.52 | **.001** |
|  |  | **Subgroup analysis per BL activity level:** |  |  |
|  |  | Change in steps per day from baseline to follow-up for sedentary group | d=0.38 | **.04** |
|  |  | Change in steps day from baseline to follow-up for low to somewhat active group | d=0.60 | **.004** |
| Evans et al 2012 [64] | Some Concerns | **Primary outcome(s):** |  |  |
|  |  | Total sitting time (hours/day [%]) | d=0.60 | .084 |
|  |  | Number of sitting events (events/day [events/hour]) | d=0.44 | **<.02** |
|  |  | Number of prolonged sitting events >30 minutes’ duration (events/day [events/hour]) | d=0.69 | **<.01** |
|  |  | Duration of prolonged sitting events (hours/day [%]) | d=0.68 | **<.007** |
| Marshall et al 2003 [62] | High | **Primary outcome(s):** |  |  |
|  |  | Change in Self-reported physical activity total (IPAQ) | d=-0.72 | *P* - not significant, values NA |
|  |  | **Secondary outcome(s):** |  |  |
|  |  | Weekday sitting | d=0.16 | *P* - not significant, values NA |
| Dadaczynski et al 2017 [61] | High | **Primary outcome(s):** |  |  |
|  |  | PA related knowledge | η^2^_p_=0.618 | **<.001** |
|  |  | PA-related intentions | η^2^_p_=0.116 | **<.001** |
|  |  | PA-related self-efficacy | η^2^_p_=0.106 | **<.001** |
|  |  | **Secondary outcome(s):** |  |  |
|  |  | Vigorous physical activity (min./week) | η^2^_p_=0.001 | .769 |
|  |  | Moderate physical activity (min./week) | η^2^_p_=0.03 | .076 |
|  |  | Minutes walked (min./week) | η^2^_p_=0.246 | **<.001** |
| Finkelstein et al 2016 [66] | Some Concerns | **Primary outcome(s)** - results for Fitbit vs control**:** | Mean difference (CI 95%) |  |
|  |  | **Moderate to vigorous PA bout min per week at 6 months** | 16 (–2 to 35) | .08 |
|  |  | Insufficiently active participants | 10 (–12 to 32) | .34 |
|  |  | Active participants | 27 (–8 to 62) | .15 |
|  |  | **Moderate to vigorous PA bout min per week at 12months** | 37 (19 to 56) | **.0001** |
|  |  | Insufficiently active participants | 24 (2 to 46) | **.04** |
|  |  | Active participants | 68 (30 to 106) | **.0005** |
|  |  | **Secondary outcome(s):** |  |  |
|  |  | Mean daily steps at 6 months | 340 (–100 to 790) | .14 |
|  |  | Mean daily steps at 12 months | 450 (–10 to 910) | .06 |
|  |  | % of participants who met the 70000 weekly step goal at 6 months | 4% (–4 to 12) | .15 |
|  |  | % of participants who met the 70000 weekly step goal at 12 months | 5% (–3 to 14) | .10 |
|  |  | Weight | No between group differences for any of the health outcomes at 6 months or 12 months. | |
|  |  | BP |  |  |
|  |  | Cardiorespiratory fitness |  |  |
|  |  | Weekly steps |  |  |
|  |  | Sedentary or light PA min/week |  |  |
|  |  | Moderate PA min/week |  |  |
|  |  | Vigorous PA min/week |  |  |
|  |  | Equivalence of MPA min/week |  |  |
|  |  | %of those meeting the 10000/day PA activity guideline |  |  |
| Thorndike et al 2014 [58] | Some Concerns | 1^st^ Phase-RCT outcomes  **Primary outcome(s):** |  |  |
|  |  | Median steps per day | IQR difference = 306 | .16 |
|  |  | Proportion of days activity monitor worn | M (%) difference = 0.2 | .73 |
|  |  | **Secondary outcome(s):** |  |  |
|  |  | Steps/day on days monitor worn | M difference = 286 | .63 |
| Urda et al 2016 [59] | High | **Primary outcome(s):** |  |  |
|  |  | Average sitting time (hours/workday) | d= -0.99 | **.012** |
|  |  | Sit-to-stand transitions (events/workday) | d=0.16 | Not significant (value not reported) |
| ***Alcohol studies*** | | | | |
| Boß et al 2017 [60] | Some Concerns | **Primary outcome(s):** |  |  |
|  |  | Weekly consumed SUA after 6 weeks (IG combined) | d=0.30 | **.001** |
|  |  | Weekly consumed SUA after 6 weeks (IG blended) | d=0.38 |  |
|  |  | Weekly consumed SUA after 6 weeks (IG pure) | d=0.25 |  |
|  |  | **Secondary outcome(s):** |  |  |
|  |  | Weekly consumed SUA after 6 months (IG combined) | d=0.42 | **.001** |
|  |  | Weekly consumed SUA after 6 months (IG blended) | d=0.38 |  |
|  |  | Weekly consumed SUA after 6 months (IG pure) | d=0.45 |  |
| Doumas & Hannah 2008 [54] | High | **Primary outcome(s):** |  |  |
|  |  | Weekend drinking (IG combined) | η2=0.07 | **.01** |
|  |  | Drinking to intoxication (IG combined) | η2=0.04 | **.05** |
|  |  | Peak consumption (IG combined) | η2=0.05 | **.05** |
|  |  | Weekend drinking (IG pure) | η2=0.14 | **.001** |
|  |  | Drinking to intoxication (IG pure) | η2=0.05 | **.05** |
|  |  | Peak consumption (IG pure) | η2=0.09 | **.01** |
| ***Multiple health behaviors studies*** | | | | |
| Cook et al 2007 [53] | High | **Primary outcome(s):** |  | *Reported that *P* was not significant |
|  |  | Attitudes towards a healthful diet | d=-0.29 | **.008** |
|  |  | Eating practices | d= 0.11 | .95 |
|  |  | Dietary stage of change | d= - 0.27 | **.01** |
|  |  | Weight Stage of Change | d= 0 | .59 |
|  |  | Godin Leisure-Time Exercise Questionnaire | d= 0.13 | NA* |
|  |  | Godin Sweat Score | d= - 0.04 | NA* |
|  |  | Exercise Behavioural Intentions | d= 0.05 | NA* |
|  |  | Exercise Self-Efficacy | d= 0.22 | NA* |
|  |  | Exercise motivation | d= -0.05 | NA* |
|  |  | Activity stage of change | d= -0.13 | NA* |
|  |  | Weight | no significant differences |  |
| Cook et al 2015 [52] | High | **Primary outcome(s):** |  |  |
|  |  | Eating practices | Δ=0.07 | 08 |
|  |  | Planning Healthy Eating | Δ=0.17 | **.03** |
|  |  | BMI | Δ=0.07 | .70 |
|  |  | Godin: Strenuous exercise | Δ=-0.11 | .61 |
|  |  | Godin: Moderate exercise | Δ=0.47 | .06 |
|  |  | Godin: Mild exercise | Δ=1.03 | **.01** |
|  |  | Godin: Sweat | Δ=0.08 | .33 |
|  |  | Godin: Overall exercise | Δ=4.98 | .08 |
|  |  | Barriers to a Healthy Diet | Δ=0.05 | .43 |
|  |  | Overeating Self-Efficacy | Δ=-0.14 | .20 |
|  |  | Diet Change Self-Efficacy | Δ=0.16 | **.05** |
|  |  | Exercise Self-Efficacy | Δ=0.08 | .11 |
|  |  | Self-Efficacy for Overcoming Barriers to Exercise | Δ=-0.68 | .78 |
|  |  | Exercise planning | Δ=0.11 | .15 |
|  |  | Tobacco Use | Analysis not performed |  |
| Deitz al 2014 [55] | High | **Primary outcome(s):** |  |  |
|  |  | Nutritional patterns | d=0.24 | .61 |
|  |  | Attitudes Toward a Healthy Diet | d=0.19 | **.003** |
|  |  | Diet Behavioral Intentions | d=0.21 | **.031** |
|  |  | Diet Behavioral Change Self-Efficacy | d=0.21 | **.015** |
|  |  | Total exercise | d=0.34 | **.016** |
|  |  | Mild exercise | d=-0.16 | .37 |
|  |  | Moderate exercise | d=-0.15 | .26 |
|  |  | Strenuous exercise | d=0.48 | **.004** |
|  |  | Exercise Self-Efficacy | d=0.35 | **.002** |
|  |  | Tobacco Abstinence Self-Efficacy Scale | d=-0.13 | **.047** |
|  |  | Weight | d=0.007 | .903 |
|  |  | Hip measurement | d=0.05 | .903 |
|  |  | Knowledge Physical | d=0.15 | .11 |
|  |  | Knowledge Behavioural | d=0.10 | .50 |
| Oftedal et al 2019 [63] | High | **Primary outcome(s):** |  |  |
|  |  | MVPA (Active Australia Q) | d=0.67 | .69 |
|  |  | Diet quality | d=0.76 | **.05** |
|  |  | Pittsburgh Sleep Quality Index | d=0.19 | .81 |

RoB – risk of bias, IG – intervention group, CG - control group, PA – physical activity, MVPA – moderate to vigorous physical activity, SUA – standard units of alcohol, NA – not reported, Statistically significant values are in **bold**
