# Supplementary material for: Effectiveness of eHealth Interventions Targeting Employee Health Behaviors: Systematic Review
Source: J Med Internet Res. 2023 Apr 20;25:e38307. doi: 10.2196/38307 (PMC10160931; doi:10.2196/38307)
Supplement: Multimedia Appendix 3 [file jmir_v25i1e38307_app3.docx]

Multimedia Appendix 3. Sample characteristics and study settings.

| Author, year, country | Sample | | | | | Setting |
| --- | --- | --- | --- | --- | --- | --- |
|  | ***N*** | ***Age***  ***M+SD*** | ***Type of participants*** | ***Female (%)*** | ***Educational attainment (%)*** |  |
| *Physical activity/sedentary behaviour* | | | | | | |
| Carr et al 2013 [51]  USA | 49 | 44.7±9.6 | Target - sedentary, overweight university employees | 90 | 78.5% college graduates | A large south-eastern university in the USA |
| Irvine et al 2011 [56]  USA | 221 | 45±9.5 | Target - Sedentary employees (PA = ≤90 min weekly) | 42.2 | College graduate 41.1%, Graduate/professional 21.7% | Large manufacturing plant. |
| Reijonsaari et al 2012 [65]  Finland | 544 | 43.5±10 | Universal: All employees | 64 | NA | Insurance company located in Helsinki, Finland |
| Slootmaker et al 2009 [67]  Netherlands | 102 | 31.8±3.5 | Target: office workers from 20 to 40 years old | 60 | 65 % highly educated | Eight worksites in the surrounding areas of Amsterdam, Netherlands |
| Poirier et al 2016 [57]  USA | 265 | 40±12 | Universal: All employees | 66 | Some college 11.3%  College graduate 46.8%  Post-graduate 37.0% | Healthways Inc - a multinational company that delivers disease management and well-being improvement solutions. |
| Evans et al 2012 [64]  UK | 30 | 44±9 | Universal: All employees | 73.3 | NA | An office at Glasgow Caledonian University, UK |
| Marshall et al 2003 [62]  Australia | 655 | 43±10 | Universal: All employees | 50 | Post-secondary education 71% | Academic (faculty) and general staff at an Australian regional university |
| Dadacyznski et al 2017 [61]  Germany | 176 | NA | Universal: All employees | 35 | 66.75% vocational training | German automobile manufacturer |
| Finkelstein et al 2016 [66]  Singapore | 800 | 35.5±8.5 | Universal: Still, mostly desk-based employees | 54 | 78% college or postgraduate education | 13 organizations spanning many industries and sectors of government in Singapore |
| Thorndike et al 2014 [58]  USA | 104 | 29 (range 23-37) | Target: physicians in training | 54 | 100% higher education (physicians) | Massachusetts General Hospital |
| Urda et al 2016 [59]  USA | 44 | 48±10 | Target: sedentary women | 100 | NA (university staff) | University in the USA |
| *Alcohol* | | | | | | |
| Boß et al 2017 [60]  Germany | 434 | 47±9.8 | Target - Risky drinkers | 59.26 | 67.56% higher education | Open recruitment in German working population. |
| Doumas. et al 2008 [54]  USA | 196 | 18-24 years old (M+SD NA) | Target - young adults 18-24 | 73 | 75% attending school (19–24-year-olds) | Five local companies in a metropolitan area in the northwest USA. |
| *Multiple health behaviours* | | | | | | |
| Cook et al 2007 [53]  USA | 419 | 42±NA | Universal – all employees | 72 | Some college: 33.65%  Bachelor’s degree: 37.45%  Postgraduate degree: 23.4% | Human resources company |
| Cook et al 2015 [52]  USA | 278 | 50-68 years old (49.6% were 50-54) | Target – employees aged 50 and over | 32.4 | Higher education: 64.7% | Global IT company |
| Deitz al. 2014 [55]  USA | 210 | NA (range 21-72) | Target: participants with at least one known risk factor for the development of cardiovascular disease. | 86 | 87% - some college experience | Three hospitals were located in Virginia, West Virginia and Ohio. |
| Oftedal et al 2019 [63]  Australia | 40 | 35.7±9.5 | Target: Shift workers | 52.5 | Mean = 16 yrs of education | Recruitment via Facebook ads for shift workers in Australia |

NA-not available or not reported
