# Supplementary material for: Effectiveness of eHealth Interventions Targeting Employee Health Behaviors: Systematic Review
Source: J Med Internet Res. 2023 Apr 20;25:e38307. doi: 10.2196/38307 (PMC10160931; doi:10.2196/38307)
Supplement: Multimedia Appendix 4 [file jmir_v25i1e38307_app4.docx]

Multimedia Appendix 4. Intervention characteristics and outcome measurement.

| **Study** | **Intervention** | **Duration of intervention** | **Mode of delivery** | **Intervention type** | **Outcome measurement** |
| --- | --- | --- | --- | --- | --- |
| ***Physical activity or sedentary behaviour interventions*** | | | | | |
| Carr et al. 2013 [51] | A multicomponent technology intervention | 12 weeks | Website  Pedometer  Pedal machine | Pure (unguided) | Activity monitor - ankle worn step watch |
| Irvine et al 2011 [56] | A stand-alone fully automated Internet-based  Intervention | 28 days | Website | Pure (unguided) | Self-report |
| Reijonsaari et al 2012 [65] | A long-term PA monitoring and counselling | 12 months | Software;  Online service (website);  Telephone counseling;  Activity monitoring device | Blended (guided) | Sickness absence data from company registry;  Self-report for PA (IPAQ) and work productivity (QQ);  Fitness test (weight, waist circumference, % of body fat, BP and aerobic fitness) |
| Slootmaker et al 2009 [67] | Web-based tailored PA advice | 3 months | Software;  Website;  Personal activity monitor | Pure (unguided) | Self-report;  Bodily composition measured objectively  Aerobic fitness test Chester Step Test with monitoring of HR |
| Poirier et al 2016 [57] | Internet-based walking program | 6 weeks | Website with gamification elements;  Activity tracker ;  Synchronization software;  Optional email/sms | Pure (unguided) | Activity tracker recorded steps |
| Evans et al 2012 [64] | Prompting software for reducing sedentary periods at work | 5 days | Educational talk;  Prompting software;  Information leaflet | Pure (unguided) | Activity monitor -activPAL |
| Marshall et al 2003 [62] | An interactive stage-targeted website and e-mail program | 8 weeks | Website;  Booklets with hyperlinks to the website;  Personalized emails | Pure (unguided) | Trained interviewers collected data via computer assisted telephone system (IPAQ) |
| Dadacyznski et al 2017 [61] | Online intervention for promoting PA in worksite settings | 6 weeks | Website (Steps and goals, Quizzes, Health goals and Challenges, gamification);  Email;  Activity tracker | Pure (unguided) | Self-report (HAPA Brief Scales, exercise self-efficacy scale, IPAQ-SF, self-developed items on PA knowledge) |
| Finkelstein et al 2016 [66] | Activity trackers with or without incentives to increase PA | 6 months | Website;  Educational booklets on strategies for increasing PA;  Activity tracker | Pure (unguided) | ActiGraph accelerometer |
| Thorndike et al 2014 [58] | Activity monitor to promote PA in physicians-in-training | Phase 1: 6 weeks | Activity tracker linked to a website;  Email;  Worksite wellness program | Pure (unguided) | Activity tracker |
| Urda et al 2016 [59] | Computer alert to get up once per hour aimed to reduce sitting time | 2 weeks | Computer alert every hour to “get up and move”;  Handout providing the options for light PA | Pure (unguided) | ActivPAL3 activity monitor |
| ***Alcohol interventions*** | | | | | |
| Boß et al 2017 [60] | Web based alcohol intervention with and without guidance | 5 weeks | IG1 – Website  IG2 – Website + eCoach | IG1: pure (unguided)  IG2: blended (guided) | Self-report |
| Doumas et al 2008 [54] | An alcohol prevention web-based personalized feedback program | IG1 - 1 session  IG2 - 1 session + motivational interview | IG1 – website  IG2 – website + MI | IG1: pure (unguided)  IG2: blended (guided) | Self-report |
| ***Multiple health behaviour interventions*** | | | | | |
| Cook et al 2007 [53] | A Web-based multimedia health promotion program | 3 months | Website | Pure (unguided) | Self-report |
| Cook et al 2015 [52] | A fully automated Web-based multimedia health promotion program | 3 months | Website | Pure (unguided) | Self-report |
| Deitz et al 2014 [55] | A Web-based health promotion program | 6 weeks | Website | Pure (unguided) | Self-report |
| Oftedal et al 2019 [63] | A mHealth intervention for improving physical activity, diet quality, and sleep quality in shift-workers | 4 weeks | Mobile phone app (Balanced app);  Move, Eat and Sleep Handbook sent via email;  Text messaging | Pure (unguided) | Self-report and interviews |

PA- physical activity, BP – Blood pressure, HR – heart rate, MI- motivational interview
